# Tracking the Trauma Epidemic in KwaZulu-Natal, South Africa

**DOI:** 10.1007/s00268-023-07032-2

**Published:** 2023-05-09

**Authors:** Aida Tefera, Elizabeth Eleanor Lutge, Nirvasha Moodley, Xolani Wiseman Xaba, Timothy Craig Hardcastle, Petra Brysiewicz, Damian Luiz Clarke

**Affiliations:** 1 Health Services Planning, Delivery, Monitoring and Evaluation Component, KZN Department of Health, Durban, South Africa; 2 School of Nursing and Public Health, University of KwaZulu-Natal, Durban, South Africa; 3 Department of Surgery, University of KwaZulu-Natal, Durban, South Africa; 4 Inkosi Albert Luthuli Central Hospital, KZN Department of Health, Durban, South Africa; 5 Department of Surgery, University of the Witwatersrand, Johannesburg, South Africa

## Abstract

**Background:**

Trauma remains an important cause of morbidity and mortality in South Africa, but attempts to track the epidemic are often based on mortality data, or derived from individual health facilities. This project is based on the routine collection of trauma data from all public health facilities in the province of KwaZulu-Natal (KZN), between 2012 and 2022.

**Methods:**

Hospital level data on trauma over the past ten years was drawn from the district health information system (DHIS). Data relating to assaults, gunshots and motor vehicle collisions (MVCs) were recorded in the emergency rooms, whilst data on admissions are recorded in the wards and intensive care units.

**Results:**

There were 1,263,847 emergency room visits for assaults, gunshots and MVCs over the ten-year period and trauma admissions ranged between four and five percent of the total number of hospital admissions annually. There was a dramatic decrease in trauma presentations and admissions over 2020/2021 as a result of the COVID lockdowns. Over the entire period, intentional injury was roughly twice as frequent as non-intentional injury. Intentional trauma had an almost equal ratio of blunt assault to penetrating assault. Gunshot-related assault increased dramatically over the 2021/2022 collecting period.

**Conclusions:**

The burden of trauma in KZN remains high. The unique feature of this burden is the excessively high rate of intentional trauma in the form of both blunt and penetrating mechanisms. Developing injury-prevention strategies to reduce the burden of interpersonal violence is more difficult than for unintentional trauma.

## Introduction

South Africa continues to experience an excessive burden of trauma. The reasons for this are complex and opaque. There can be little doubt that poverty, poor governance, high levels of criminality, ready availability of alcohol, increased rates of urbanization and motor vehicle usage all contribute to this pandemic. Despite this, there is very little in the way of a co-ordinated attempt to track and monitor this pandemic. KwaZulu-Natal is the second most populous province in the country and covers a large and varied geographical area, ranging from mountain ranges in the west to subtropical coastal areas in the east and the north. There is a huge burden of trauma in KZN Province [1]. This burden of trauma interacts with the high levels of social deprivation and poverty which afflict the province. In 2012, a program was initiated to track the burden of trauma in KZN by adding trauma to the mandatory institutional monthly reporting system known as the District Health Information System (DHIS). This system tracks admission and transfer data for several diseases. Trauma was included on this system in KwaZulu-Natal in April 2012. Trauma-specific datasets were chosen which would be both practical to collect and which would provide useful data. The selected datasets included: all patients seen in the emergency room with a diagnosis of trauma due to blunt assault, motor vehicle collisions (MVC), stab, and gunshot wound. The initial review of this system was performed in 2015 and yielded useful information which was published in the literature [2]. The system has now been running for a decade and this current project sets out to review the cumulated data.

## Material and methods

The project was formally launched in April 2012. The hospital-level DHIS data on trauma from 1st April 2012 to 31st March 2022 is reviewed in this report. All forty-seven provincial public sector hospitals at the district (33), or regional/tertiary (13) level and the single central level facility were included in this analysis. The data from the DHIS are aggregated at facility level and so provided in the form of counts (total patient numbers) without patient identifiers, thus prohibiting data-linkage between the data collected in the emergency room, in the wards, and in the intensive care units (ICUs). Data relating to the cause of the trauma (blunt assaults, stabs, gunshots and MVCs) are recorded in the emergency rooms whilst data on admissions are recorded in the wards and ICUs. During the first eight years of this period, assault data were disaggregated into assault with blunt and sharp instruments, and MVC data into pedestrians and occupants of vehicles. In the last two years of review, the data collection was simplified into assault and MVC only. Gunshots were collected as a distinct group throughout the period. Ethics approval to analyse data from the DHIS was obtained from the Biomedical Research Committee of the University of KwaZulu-Natal (reference BCA056/13).

The “trauma system” in KwaZulu-Natal functions in the dichotomous private–public system. There is a large (underfunded) public health system that caters to the largely indigent 85% of the population and a private fee-for-service sector for those with medical insurance. Emergency Medical Services share this dichotomy; however, they are obligated to treat and transport trauma victims to the nearest appropriate facility in the public sector. Prehospital care is independent practice and has three levels of care. There is a developing aeromedical capacity. The district facilities are largely Family Medicine and general practitioner staffed, with limited major trauma capacity [3]. The regional and tertiary facilities have general surgeons, orthopaedic surgeons and anaesthetists, who will all have done ATLS™ prior to qualification, and who are capable of emergency surgical care, albeit with limited access to operation rooms and ICU capacity. The teaching tertiary facilities and the quaternary facility have sub-specialist trauma surgeons with intensive care training. Neurosurgery and vascular surgery are only to be found at the one tertiary hospital and the quaternary facility. Often the shortage of intensive care capacity will result in inter-hospital transfers post-emergency surgery for definitive care [4, 5]. Post-discharge rehabilitation services are severely challenged [6].

## Data analysis

Simple count data, aggregated to provincial level, were presented and described. Counts were also related to the provincial population and presented as rates per unit population. Where statistical analysis was done, Stata MP version 17 for Windows [Statacorp, College Station, TX] was used to analyse the data. Data were aggregated at the year level. Poisson regression analysis for count data was used to assess the incidence rate ratio of each successive year relative to the baseline year of 2012. The number of incidents was used as the dependent variable, the year as the independent variable (specified as an indicator variable) and the exposure variable was the denominator in each case (for example, total population at each year). The incidence rate ratio was indicated for each year relative to the reference year of 2012. Also reported are 99% confidence intervals. This is a method of using all the available population level data even when it is aggregated at the year level, thereby increasing statistical power.

## Results

**Fig. 1:**
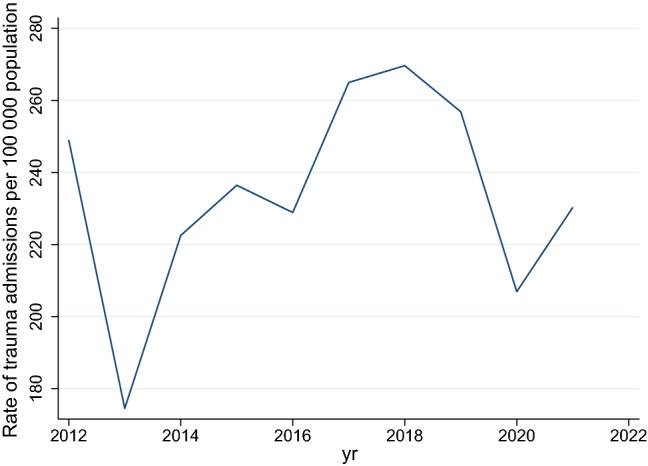
Rate of trauma per 100,000 population over time. (The initial decline is believed to be due to collection problems at the onset of data collection)

There were a total of 1 263 847 visits to the emergency rooms for trauma over the ten-year period. These ranged from a high of 134 550 in 2017/18 to a low of 104 969 in 2020/2021. This is demonstrated in Fig. 1 and Table 1. The ratio of intentional/assault injury to non-intentional/MVC injury ranged between 1.7:1 and 2:1 over the period. The ratio of blunt to penetrating assault was roughly equal over the entire period. The ratio of MVC occupants to MVC pedestrians was in the order of 2.3:1. The absolute number of MVC-related presentations increased over the decade but showed a dramatic decrease in 2020/2021. This is demonstrated in Table 1.

**Table 1:** Trauma-related emergency room visits to public sector hospitals in KwaZulu-Natal

	2012/2013	2013/2014	2014/2015	2015/2016	2016/2017	2017/2018	2018/2019	2019/2020	2020/2021	2021/2022
Non-Intentional/MVC	40,412	43,000	40,633	45,201	49,431	49,890	50,529	50,799	35,165	43,838
Intentional/assault^a^	67,734	87,092	80,304	82,899	84,355	84,660	83,776	87,317	69,804	87,008
Ratio intentional/assault to non-intentional/MVC	1.7	2.0	2.0	1.8	1.7	1.7	1.7	1.7	2.0	2.0
Assault blunt	33,885	45,794	39,983	42,670	42,746	43,414	42,856	43,347		
Assault stabs	32,866	37,584	36,513	36,275	37,317	36,798	36,221	39,476		
Ratio assault blunt: assault stab	1.0	1.2	1.1	1.2	1.1	1.2	1.2	1.1		
Total assault (blunt plus stab)	66,751	83,378	76,496	78,945	80,063	80,212	79,077	82,823	65,070	78,522
GSW	983	3714	3808	3954	4292	4448	4699	4494	4734	8486
MVC total	40,412	43,000	40,633	45,201	49,431	49,890	50,529	50,799	35,165	43,838
MVC Occupants	27,015	29,804	28,186	32,456	35,367	35,289	35,436	35,046		
MVC Pedestrian	13,397	13,196	12,447	12,745	14,064	14,601	15,093	15,753		
Ratio MVC occupant: MVC pedestrian	2.0	2.3	2.3	2.5	2.5	2.4	2.3	2.2		

^a^Assault blunt plus assault stab plus gunshots

The total number of admissions in public hospitals in KZN and the total number of trauma admissions per year are outlined in Table 2. There has been a gradual decline in total hospital admissions over the decade, which has been more pronounced over the last three years (Table 2). The total number of trauma admissions showed an increasing trend from 2014/15, and then declined during the last three years of review. Out of the total admissions, the percentage of trauma patients shows an increase between 2012 and 2022. Over the past six years, trauma admissions ranged between four and five percentage of the total number of hospital admissions annually.

**Table 2:** Admissions to public sector hospitals in KwaZulu-Natal 2012/13 to 2021/22

	2012/2013	2013/2014	2014/2015	2015/2016	2016/2017	2017/2018	2018/2019	2019/2020	2020/2021	2021/2022
Total hospital admissions	682,818	679,379	679,752	657,981	630,399	632,496	629,395	639,124	547,367	579,304
Trauma admissions	25,959	18,479	23,925	25,816	25,373	29,816	30,794	29,744	24,298	27,316
Percentage trauma admissions	3.8	2.7	3.5	3.9	4.0	4.7	4.9	4.7	4.4	4.7

**Fig. 2:**
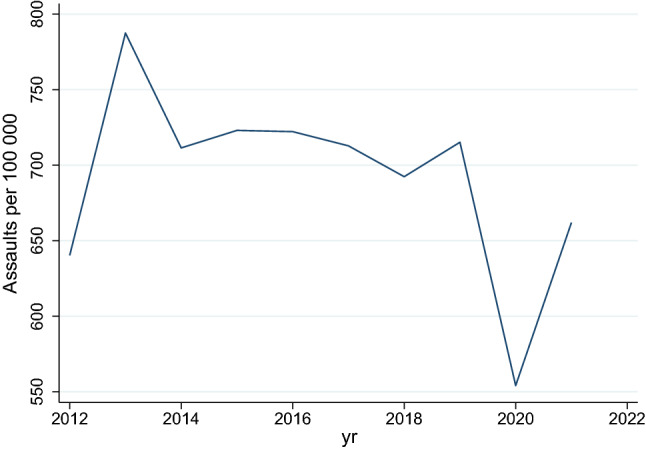
Rate of assault per 100,000 population over time

**Fig. 3:**
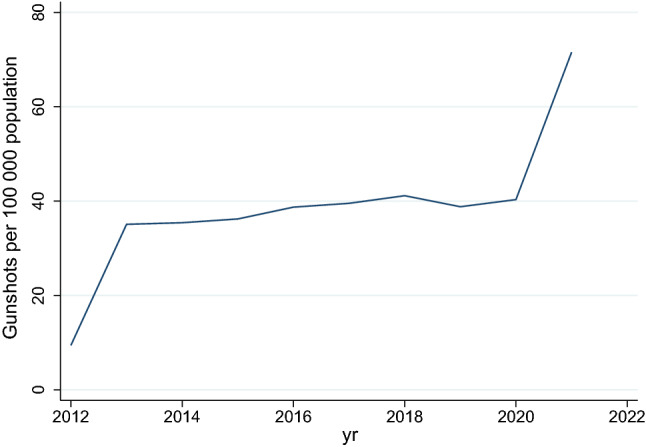
Rate of gunshot injury per 100,000 population

The Poisson model showed that there was a negative incidence rate of trauma admissions per 100 000 population in years 2013 to 2016 relative to the baseline year of 2012, whereafter the rate increased from 2017 to 2019 and then fell below the 2012 rate in 2020 and 2021. This is shown in Fig. 1. The rate of assault increased by 23% between 2012 and 2013, and remained steady between 2014 and 2019, whereafter it dropped in 2020 by 13.5% of the rate it was in 2012 and then increased to just over baseline level in 2021. This is shown in Fig. 2. The ratio of rates of assault with a sharp instrument to those with a blunt instrument remained stable over the period. The absolute number and rate of injury due to gunshots increased from 2012 to 2013 and remained steady until 2020, whereafter it increased in 2020/2021 (see Table 3). In 2021, the incidence rate of gunshots was 7.6 times higher than it was in 2012. This is illustrated in Fig. 3.

**Table 3:** Rates of injured patients presenting to the emergency room per 100 000 population in KZN

	Apr 2012 to Mar 2013	Apr 2013 to Mar 2014	Apr 2014 to Mar 2015	Apr 2015 to Mar 2016	Apr 2016 to Mar 2017	Apr 2017 to Mar 2018	Apr 2018 to Mar 2019	Apr 2019 to Mar 2020	Apr 2020 to Mar 2021	Apr 2021 to Mar 2022
Trauma admissions	249.0	174.5	222.5	236.4	228.9	265.0	269.6	256.8	206.9	230.3
Gunshots	9.4	35.1	35.4	36.2	38.7	39.5	41.1	38.8	40.3	71.5
Assaults	640.3	787.5	711.4	723.0	722.2	712.8	692.4	715.2	554.1	662.0
MVC injuries	387.6	406.1	377.9	414.0	445.9	443.3	442.4	438.6	299.5	369.6

## Discussion

These data highlight a number of features which distinguish the trauma burden in KZN and South Africa from other regions. The absolute rate of trauma in KZN has remained persistently high over the last decade and the absolute number of both emergency room visits and trauma admissions has increased over the decade. The proportion of hospital admissions due to trauma increased over the period, approaching 5% in the last five years of the decade under review.

The next striking and distinguishing feature is the fact that intentional/assault trauma exceeds unintentional/MVC trauma by a ratio of 2:1. Globally, the majority of deaths due to trauma in 2013 resulted from unintentional injury such as MVC’s (29.1%), self-harm (17.6%) and falls (11.6%) [7]. Interpersonal violence accounted for only 8.5% of world-wide reported trauma-related deaths. In contrast, in this study, the rate of interpersonal violence and intentional injury dramatically exceeded that of unintentional injury by a ratio of two to one. This finding is echoed in recent South African reports from both a regional [8] and tertiary [9] hospital in KZN. This distinguishes trauma in South Africa from other parts of the continent and the world. In a study of a regional hospital in Tanzania, MVC’s were the cause of 60% of all emergency room visits [10]. The major European causes of injury are MVC’s, falls and self-harm [11]. In KZN, the rates of emergency room attendance for assault/intentional trauma are significantly higher than for non-intentional trauma [1, 2]. Another unique feature is that the ratio of assault with a sharp instrument compared to assault with a blunt instrument was approximately 1:1 over the period. Once again, this contrasts with reports from other regions, where blunt injury is responsible for the vast majority of assaults/intentional trauma [12].

Injury-prevention programs in South Africa need to target both intentional and non-intentional injury. Non-intentional injury secondary to MVC’s can be prevented by mechanical interventions such as improved road safety, traffic calming and separating pedestrians from traffic. These strategies are effective and relatively easy to implement. The ratio of MVC-related occupants to pedestrians is 2:1. This implies that pedestrians are exposed to traffic and are vulnerable to being injured by vehicles. This highlights the lack of pedestrian protection in the country. Barriers to separate pedestrians from traffic, pedestrian exclusive areas, traffic calming, and pedestrian bridges are designed to keep pedestrians and traffic separate [13]. Attention needs to be paid to structural design in urban planning and road building to achieve this. Adequate policing of road laws will also assist; however, the police are also under-resourced [14].

Reducing rates of intentional trauma are difficult as prevention strategies must attempt to modify individual human behaviour and the moral compass of the community [15]. The COVID pandemic and the social interventions implemented in response to the pandemic, dramatically reduced trauma admissions. A comparison of emergency room data for a regional hospital in KwaZulu-Natal showed a 47% reduction in trauma cases in April 2020 compared to the preceding two years [16]. This affected both MVC/non-intentional trauma and intentional/assault-related trauma. This reduction is mirrored in the USA [17] and Australia [18]. However, the absolute number of trauma admissions, as well as the rate of trauma admissions per 100 000 population, reverted to pre-COVID levels in 2021/22. This quick return to pre-COVID levels was also seen in the USA [19]. This implies that draconian interventions and restrictions on personal freedoms can temporarily reduce the absolute number of trauma cases. However, once these restrictions are lifted, trauma rates rapidly return to pre-restriction levels. The exception to this decrease in trauma during the COVID pandemic was the absolute number of gunshot-related injuries. The reasons for the dramatic increase in gunshot-related injury over the last decade are unclear and may reflect ongoing political instability and increasing crime levels.

Trauma in SA remains a neglected epidemic. Whilst the infective epidemics of HIV and TB are extensively studied from multiple academic perspectives, the same cannot be said about trauma. This needs to change and there needs to be a move into advocacy and partnership with non-governmental organizations and charities, as well as with other academic disciplines such as sociology, in order to reduce the burden of trauma. The focus over the last decade has been on the development of a provincial trauma system. This has resulted in a nascent system with an established trauma training program and with two level one trauma centres in KZN. There are also early signs that the private and public sector trauma systems are beginning to achieve a degree of synergy. The next step in developing this comprehensive system is to increase community engagement around issues of prevention of violence. Injury-prevention strategies must be tailored to the local environment, and this requires cultural sensitivity and community buy-in [20]. A more defined legislated quality-assured trauma system, with adequate funding, an integrated prehospital component and post-discharge rehabilitation remains the objective and further research utilizing the three-delays system may give better directions for system change [21].

## Conclusion

The burden of trauma in KZN remains high. The unique feature of this burden is the excessively high rate of intentional trauma in the form of both blunt and penetrating mechanisms. Developing injury-prevention strategies to reduce the burden of interpersonal violence is more difficult than for unintentional trauma. The draconian limitations on personal freedom imposed during the COVID lockdowns reduced all trauma-related admissions.

## References

[1] Hardcastle T, Oosthuizen G, Clarke D, Lutge E, Padarath A, King J, Mackie E, Casciola J (2016). Trauma, a preventable burden of disease in South Africa: review of the evidence, with a focus on KwaZulu-Natal. South African health review.

[2] Lutge E, Moodley N, Tefera A (2016). A hospital based surveillance system to assess the burden of trauma in KwaZulu-Natal Province South Africa. Injury.

[3] Clarke DL, Aldous C, Thomson SR (2014). Assessing the gap between the acute trauma workload and the capacity of a single rural health district in South Africa. What are the implications for systems planning?. Eur J Trauma Emerg Surg.

[4] Hardcastle TC, Brysiewicz P (2013). Trauma in South Africa: from humble beginnings to an Afrocentric outreach. Int Emerg Nurs.

[5] Zhou J, Wang T, Belenkiy I, Hardcastle TC, Rouby JJ, Jiang B, for the International Trauma Rescue & Treatment Association (ITRTA) Study Group (2021). Management of severe trauma worldwide. Implementation of trauma systems in emerging countries: China, Russia and South Africa. Crit Care Med 25: 286 doi: 10.1186/s13054-021-03681-8 10.1186/s13054-021-03681-8 PMC8352140 34372903

[6] Hardcastle TC (2021). Trauma rehabilitation services in low- and middle-income countries – the challenge to human recovery. Adv Hum Biol.

[7] Haagsma JA, Graetz N, Bolliger I (2016). The global burden of injury: incidence, mortality, disability-adjusted life years and time trends from the global burden of disease study 2013. Inj Prev.

[8] Lewis C, Wood E (2015). Interpersonal violence as a major contributor towards the skewed burden of trauma in KwaZulu-Natal, South Africa. S Afr Med J.

[9] Donovan MM, Kong VY, Bruce JL (2019). The hybrid electronic medical registry allows benchmarking of quality of trauma care: a five-year temporal overview of the trauma burden at a major trauma centre in South Africa. World J Surg.

[10] Sawe HR, Wallis LA, Weber EJ (2020). The burden of trauma in Tanzania: analysis of prospective trauma registry data at regional hospitals in Tanzania. Injury.

[11] Haagsma JA, Charalampous P, Ariani F (2022). The burden of injury in central, eastern, and western European sub-region: a systematic analysis from the global burden of disease 2019 study. Arch Public Health.

[12] Difino M, Bini R, Reitano E (2021). Epidemiology of trauma admissions in a level 1 trauma center in Northern Italy: a nine-year study. Updates Surg.

[13] Forjuoh SN (2003). Traffic-related injury prevention interventions for low-income countries. Inj Control Saf Promot.

[14] Bezuidenhout C, Kempen A, Albrecht JF, den Heyer G (2021). Historical and current dilemmas in South Africa that challenge proficient police service delivery. Enhancing police service delivery.

[15] Van der Walt JL (2019). The search for a moral compass and a new social contract in the context of citizenship education. HTS Teologiese Studies/Theol Stud.

[16] Morris D, Rogers M, Kissmer N (2020). Impact of lockdown measures implemented during the Covid-19 pandemic on the burden of trauma presentations to a regional emergency department in Kwa-Zulu Natal, South Africa. Afr J Emerg Med.

[17] Kamine TH, Rembisz A, Barron RJ et al (2020). Decrease in trauma admissions with COVID-19 Pandemic. West J Emerg Med 21(4): 819–822. doi: 10.5811/westjem.2020.5.47780. PMID: 32726250; PMCID: PMC7390569 10.5811/westjem.2020.5.47780 PMC7390569 32726250

[18] Jacob S, Mwagiru D, Thakur I (2020). Impact of societal restrictions and lockdown on trauma admissions during the COVID-19 pandemic: a single-centre cross-sectional observational study. ANZ J Surg.

[19] Ghafil C, Matsushima K, Ding L (2021). Trends in trauma admissions during the COVID-19 pandemic in Los Angeles county California. JAMA Netw Open.

[20] Amisi MM, Naicker SN. (2021) Review: an evidence review of violence prevention in South Africa. Institute for Security Studies. Available at: https://issafrica.s3.amazonaws.com/site/uploads/policybrief162.pdf (accessed 14/032023)

[21] Whitaker J, O'Donohoe N, Denning M (2021). Assessing trauma care systems in low-income and middle-income countries: a systematic review and evidence synthesis mapping the three delays framework to injury health system assessments. BMJ Glob Health.

